# 728 Chemical Burns and Eyebrow Tinting: An Unusual Case

**DOI:** 10.1093/jbcr/irac012.282

**Published:** 2022-03-23

**Authors:** Kimberley C Brondeel, Robert P Duggan, Katie Kirk, Alen Palackic, Ludwik K Branski

**Affiliations:** University of Texas Medical Branch, Galveston, Texas; University of Texas Medical Branch, Galveston, Texas; University of Texas Medical Branch, Galveston, Texas; University of Texas Medical Branch, Galveston, Texas; University of Texas Medical Branch, Galveston, Texas

## Abstract

**Introduction:**

Here we present the case of chemical burns following professional eyebrow tinting, a phenomenon rarely described in the literature.

**Methods:**

A 50-year-old previously healthy female presented to our emergency department for evaluation and treatment of chemical burns to her eyebrows. Fifteen days prior, she underwent professional eyebrow tinting by a local esthetician. Blistering developed the following day, and progressive swelling prompted her to present to an outside hospital. There she was prescribed acyclovir, ketoconazole cream, and silver sulfadiazine, but despite these measures, her symptoms progressed, leading to her presentation to our facility. Both brows were remarkable for significant swelling with exudative crushing on the surface concerning for superficial infection of partial-thickness chemical burns. She was prescribed bacitracin, prednisone, and clindamycin and discharged with instructions to follow up in burn clinic. Six days later, both brows had developed 2x3 cm scabs, but no signs of infection were appreciated. She was instructed to apply bacitracin/polymyxin B ointment to the scabbing areas and open wounds. Forty-nine days after her tinting, both brows were noted to be completely healed with no alopecia.

**Results:**

Burns following cosmetic procedures most commonly occur during hair lightening treatments where products frequently contain caustic chemicals such a hydrogen peroxide or persulphates leading to oxidation reactions lightening the hair. Thermal burns in hair salons have also been reported to heated hair-dressing instruments or external heat to hasten the highlighting process.

The periorbital area is becoming an increasingly popular target for nonsurgical cosmetic procedures, including permanent eyelid tattooing, eyelash dying, and extensions, more recently, eyebrow tinting. Eyebrow tinting involves the application of semipermanent dyes to give the appearance of a fuller brow. There are no FDA-approved brow tinting formulations, and many contain para-phenylenediamine (PPD), a dying agent frequently associated with allergic dermatitis and less commonly chemical burns. This patient underwent brow tinting in a professional setting and still experienced a chemical burn. As the popularity of brow tinting increases, some patients will undoubtfully seek out readily available, unregulated products for self-application. The potential for dermatitis and chemical burns following eyebrow tinting will only increase.

**Conclusions:**

Eyebrow tinting is not a benign cosmetic procedure, and even professional application of dyes may lead to chemical burns. We believe an increased awareness of brow tinting and its potential complications is warranted given its increasing popularity, the preponderance of unregulated products, and the potential for poor cosmetic outcomes.

